# Extraction of Galphimines from *Galphimia glauca* with Supercritical Carbon Dioxide

**DOI:** 10.3390/molecules25030477

**Published:** 2020-01-22

**Authors:** Francisco Javier Verónico Sánchez, Octavio Elizalde Solis, Alejandro Zamilpa, Ricardo García Morales, Ma. Dolores Pérez García, Jesús E. Jiménez Ferrer, Jaime Tortoriello

**Affiliations:** 1 Departamento de Ingeniería Química Petrolera and Sección de Estudios de Posgrado e Investigación, Escuela Superior de Ingeniería Química e Industrias Extractivas, Instituto Politécnico Nacional (IPN), Mexico City 07738, Mexico; javi.ibq@hotmail.com (F.J.V.S.);; 2 Centro de Investigación Biomédica del Sur, Instituto Mexicano del Seguro Social (CIBIS-IMSS), Morelos 62790, Mexicojaime.tortoriello@imss.gob.mx (J.T.)

**Keywords:** *Galphimia glauca*, galphimine B, árnica roja, supercritical extraction, carbon dioxide, kinetic modeling

## Abstract

The anti-depressive and anxiolytic effect of galphimine B (isolated from *Galphimia glauca*) has been demonstrated by researchers. Therefore, it is necessary to explore extraction techniques that produce materials with adequate quality for pharmaceutical applications. In this work, supercritical extractions of galphimines from *Galphimia glauca* were performed in the presence of carbon dioxide. Pressure, temperature, particle diameter, and flow rate effects were examined to explore the conditions with the highest yield and the concentration profile of galphimines in the studied interval. The identification of the nor-seco triterpenoids and galphimine B and E was carried out by HPLC analyses. The mathematical modeling of the extraction curves was attained by the approaches proposed by Sovová and Papamichail et al. According to results, the highest yield 2.22% was obtained at 323.15 K, 326 μm, 3 L/min, and 33.75 MPa. Meanwhile, the content of galphimine B in the extract was, on average, 19.5 mg·g^−1^.

## 1. Introduction

*Galphimia glauca* is the scientific name for the plant popularly known in Mexico as “arnica roja”. This species has oval leaves, yellow flowers, and small capsule-shape fruits. Traditional medicine attributes its healing properties for the treatment of wounds and rash, postpartum issues, antirheumatic properties, and nervous calming [1,2]. Regarding scientific research, the biological activity of chemical compounds constituted in several plants is reported in some paper reviews; *Galphimia glauca* is included in these databases [3,4,5].

**Figure 1:**
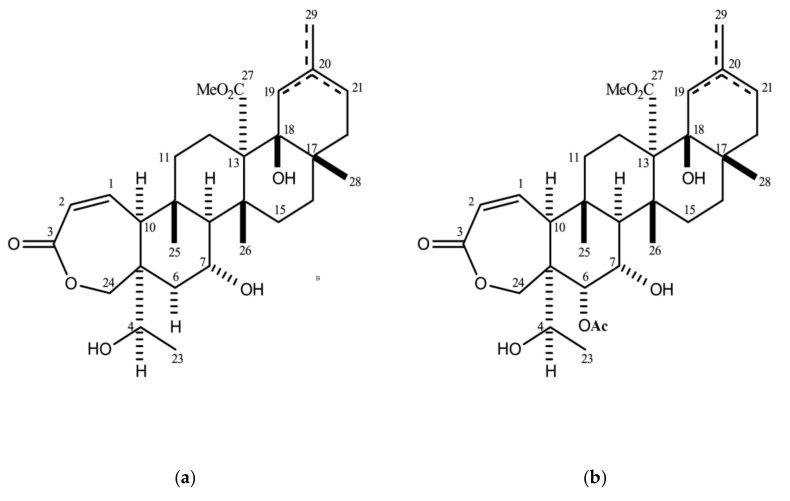
Molecular structures of galphimines: (**a**) galphimine B; (**b**) galphimine E [14].

The biological activity attributed to chemicals in *Galphimia glauca* has been mainly demonstrated as an antioxidant [6], anti-inflammatory [7], anxiolytic, and sedative [2]. Besides, some alkanes, alkenes, carboxylic acids, aliphatic alcohols, terpenes, and terpenoids have been identified in extracts from *Galphimia glauca* by means of characterization techniques [7]. Among these compounds, there is a family of nor-seco triterpenoids called galphimines, whose extraction and biological activity are fully described elsewhere [8,9]. Two of the most important constituents in galphimines are galphimine B and galphimine E, whose isolation and characterization have been explained elsewhere for pharmaceutical applications [10]. The therapeutic effect of the extract from *Galphimia glauca* (from 1.5 to 53 mg galphimine B/g extract) has been successfully proved. It was administered as medicine for anxiety treatment in young adults. According to the reported results, no significant statistical difference was found between people treated with this extract and people prescribed with sertraline and lorazepam [11,12,13]. These pharmaceutical drugs are approved as anti-depressive and anxiolytic. Figure 1 shows the structures of galphimine B (G-B) and galphimine E (G-E) [14].

For extraction purposes, some of the common organic solvents that have been used for obtaining natural extracts are ethanol, water, methanol, and petroleum ether [6,15]. Conventional extraction procedures using these solvents achieve high yields (from 5.1% to 25%, [11,12,13]), but the separation and purification stages may be complicated. Besides, some of the extracted compounds may suffer from thermal degradation if high temperatures are employed [16].

On the other hand, extraction via supercritical fluids is an alternative technique to extract substances from an insoluble matrix. The properties of supercritical fluids make them suitable mixing with solutes and transporting this fluid mixture out from a matrix faster than organic solvents as its solvation power is like liquids, and diffusivity is like gases. Another advantage is the absence of solvent residues in the obtained extracts. The most common supercritical fluid used for extraction is carbon dioxide due to its moderate critical properties, chemical stability, inertness, inflammability, being non-toxic, and harmless to the environment. Carbon dioxide can be obtained as a byproduct of fermentation, combustion, and ammonia synthesis. This fluid can also be recycled in supercritical extractions [17].

In particular, knowledge of mass transfer phenomena and adequate mathematical modeling are essential for a proper design of a supercritical fluid extraction (SFE) process. The design includes scaling up and economic feasibility [18]. For the modeling and analysis of the extraction curves, the developed models usually require thermodynamic and kinetic variables: density, viscosity, diffusivity, mass transfer coefficients, axial dispersion, and solubility. In these models, yield (often defined as the mass ratio between extract and the raw material loaded in the extractor) is the dependent variable, while time is the independent variable. The yield of SFE is influenced by some variables and can be globally enclosed in pressure and temperature. Both variables make changes in density and solvation power of carbon dioxide; the effect is even more remarkable in the vicinity of its critical condition. Solvent flow rate, as well as particle diameter from the matrix, also influences the extraction yield due to mass transfer phenomena involved during the SFE process [18].

The models published in the literature are based on a material balance in a particle and the fluid during the extraction; hence, they share similar assumptions [19,20,21,22,23]. One of the most applied models in SFE is the one proposed by Sovová [24], which considers the milling of a sample matrix that produces broken and intact cells. It is important to note that Sovová’s model does not take into account solute-matrix interactions because it was originally developed for vegetable oil extracts from seeds. Despite the above-mentioned assumption, this model has been successfully applied in other kinds of extracts [25]. Another important approach has been proposed by Papamichail et al. [26], which considers the solute-matrix interactions that are present in several SFE from plants.

To the best of our knowledge, there is no documented research about supercritical fluid extraction of natural products from *Galphimia glauca*, nor information about the solubility of galphimines in solvents (either liquid or supercritical solvents). With this background, the aim of this work was to perform supercritical extractions of galphimines from *Galphimia glauca* using carbon dioxide at different conditions to evaluate the influence of pressure, temperature, particle diameter, and flow rate. The analyses for the composition of the extracts were carried out with the HPLC technique. The approaches proposed by Sovová and Papamichail et al. were also evaluated in the modeling of the extraction kinetics.

## 2. Results and Discussion

### 2.1. Extraction Behavior

The influence of pressure (*P*), temperature (*T*), particle diameter (*d*_p_), and volumetric flow rate (*Q_V_*) over the supercritical fluid extraction of *Galphimia glauca* was evaluated in terms of the extraction yield (*e*), defined as the ratio between the mass of extract and the mass of solid material fed into the extractor. Experimental conditions were set in a way that a variable was changed in proportional increments, while the others were fixed at a central value. There were four levels for each variable. The extraction yield reached a maximum of 2.22%, although the extraction experiments did not include a static period that would promote better contact between *Galphimia glauca* and carbon dioxide at the beginning.

The maximum yield of 2.22% obtained from *Galphimia glauca* was lower than those obtained from materials (*e* ≤ 50%) that are not herbaceous, such as seeds [27] or animal wastes [28], which are constituted mainly by fats. In contrast, the yields (*e* < 2.22%) were comparable with *e* < 7% obtained from other plants using supercritical carbon dioxide extractions [29,30,31]. The probable presence of several compounds, such as non-polar (alkanes, monoterpenes) [32,33], slightly polar (phenolics, carboxylic acids, ketones, terpenoids) [34,35,36,37], and fats (waxes, esters) [38,39], in the *Galphimia glauca* extract, might affect the yield. These compound families have been found in supercritical carbon dioxide extracts of several plant materials, using aerial parts like this study.

**Figure 2:**
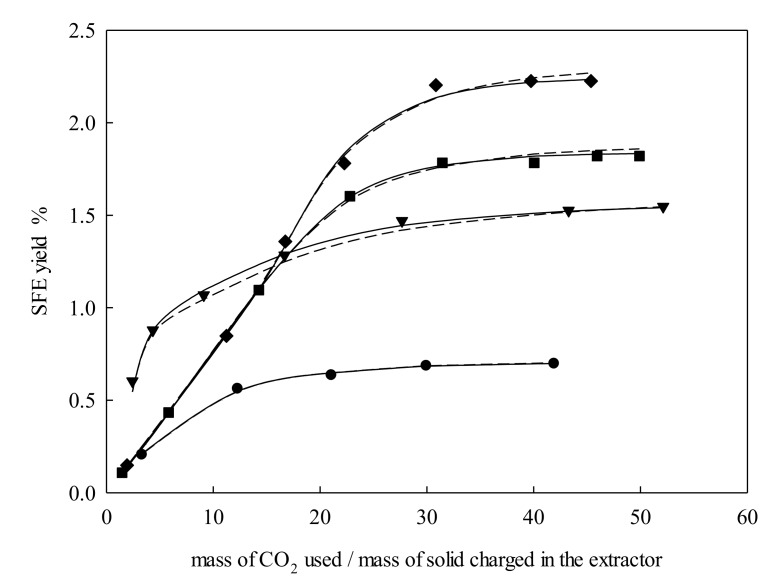
Supercritical fluid extraction curves of *Galphimia glauca* at temperature = 323.15 K, particle size = 326 μm, and volumetric flow rate = 3 L·min^−1^: ●, 15.00 MPa; ▼, 21.25 MPa; ■, 27.50 MPa; ◆, 33.75 MPa. Lines denote the modeling: –––, Sovová [24]; – – –, Papamichail et al. [26].

It was found that *e* was directly proportional to pressure changes by keeping *T*, *d*_p_, and *Q_V_* constant, as depicted in Figure 2. This must be attributed to the increase in density that went from 699.75 kg·m^−3^ (15 MPa) to 892.55 kg·m^−3^ (33.75 MPa). The latter condition enhanced the solvating power of the supercritical fluid and dissolved more solutes. Besides, the solubility of solutes in supercritical fluids tended to increase with pressure, leading to an increase in *e*.

**Figure 3:**
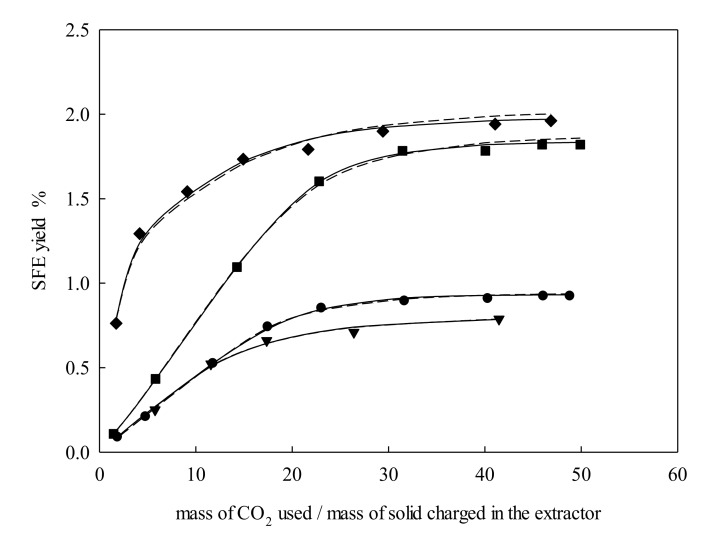
Supercritical fluid extraction curves of *Galphimia glauca* at *P* pressure = 27.50 MPa, particle size = 326 μm, and volumetric flow rate = 3 L·min^−1^: ●, 313.15 K; ▼, 318.15 K; ■, 323.15 K; ◆, 328.15 K. Lines denote the modeling: –––, Sovová [24]; – – –, Papamichail et al. [26]

The temperature effect in the supercritical fluid extraction yield could be indirectly analyzed as the influence of carbon dioxide density and the vapor pressure of the solute(s) under study. The results illustrated in Figure 3 showed that *e* increased, while temperature was also increasing at fixed *P*, *d*_p_, and *Q_V_*, except for the increment from 313.15 K to 318.15 K. This increment of yield might be attributed to a better dissolution of the solutes because of their vapor pressure increment at a high temperature. This hypothesis could be further tested by measuring the solubility of the extract in supercritical carbon dioxide in order to check the most dominant effect in solubility.

**Figure 4:**
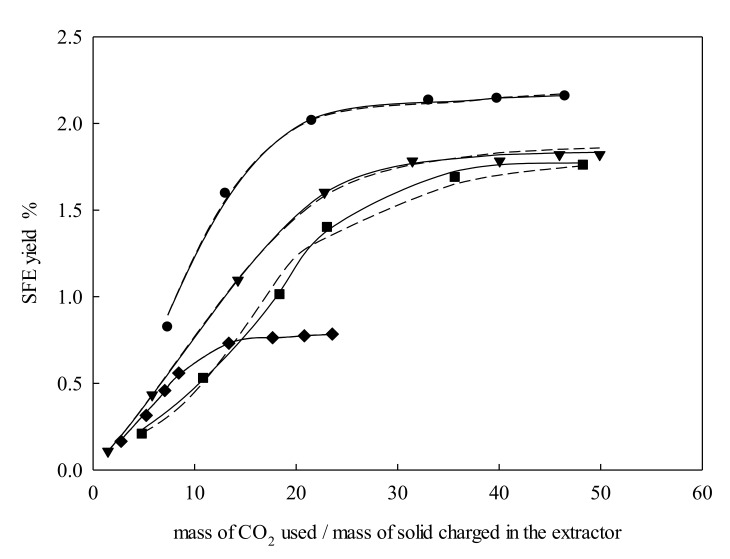
Supercritical fluid extraction curves of *Galphimia glauca* at pressure = 27.50 MPa, temperature = 323.15 K, and volumetric flow rate = 3 L·min^−1^: ●, 224 μm; ▼, 326 μm; ■, 461 μm; ◆, 548 μm. Lines denote the modeling: –––, Sovová [24]; – – –, Papamichail et al. [26]

The influence of particle size variations is shown in Figure 4. The extraction yield tended to increase when the particle size was small. The reduction of particle size expanded the interfacial area, as well as released the solutes from the cell structures found in the plant. As the experiments plotted in Figure 3 were carried out at the same *P*, *T,* and *Q_V_* conditions, the first part of the extraction curves was quite similar.

**Figure 5:**
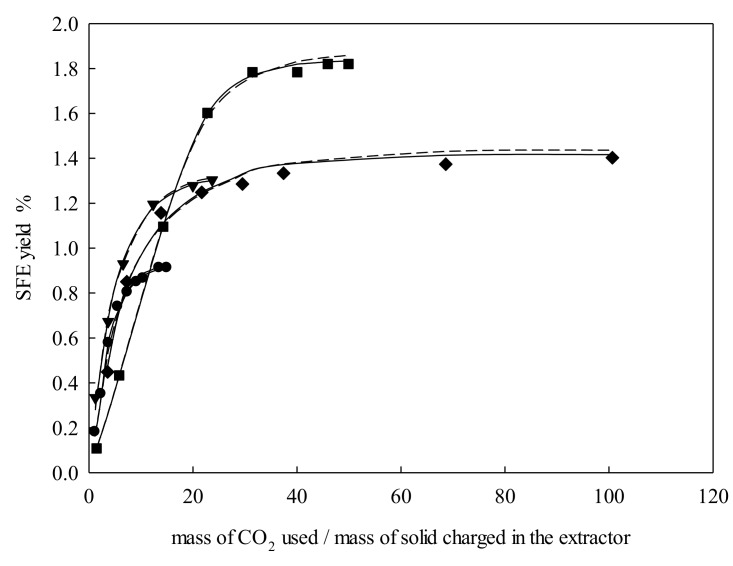
Supercritical fluid extraction curves of *Galphimia glauca* at pressure = 27.50 MPa, temperature = 323.15 K, and particle size = 326 μm: ●, 1 L·min^−1^; ▼, 2 L·min^−1^; ■, 3 L·min^−1^; ◆, 4 L·min^−1^. Lines denote the modeling: –––, Sovová [24]; – – –, Papamichail et al. [26]

Regarding the volumetric flow rate, the behavior of this variable could be observed in Figure 5. The first part of the extraction curve had the same performance as the corresponding section for the particle size analyses. It indicated that the carbon dioxide was saturated with the extract at the stated flow. Yield values were increasing until the volumetric flow rate reached 3 L·min^–1^, but *e* values decreased after this rate. Probably, the low residence time avoided the solute solubilization in the supercritical fluid at the highest flow rate.

### 2.2. Modeling

In general, the modeling with pressure variations produced similar results regarding the parameters from both proposals, as could be observed in Table S1. External mass transfer coefficients for the Sovová model decreased while pressure was increasing. This trend was due to the increase in carbon dioxide density, which consequently decreased the solvent velocity and the mass transfer rate [25]. The external mass transfer coefficient was one order of magnitude higher than the internal one. This indicated that convection controlled the process rather than intraparticle diffusion. The same trend was kept for the overall mass transfer coefficients from the model of Papamichail et al. These parameters tended to decrease with pressure increasing. The opposite was observed for transition periods for each model based on pressure changes, which this time had the tendency to increase as pressure rose. This trend was the opposite of what has been reported in other extractions of plant materials [40,41]. The behavior of the equilibrium constant respect to pressure variations followed the same trend of Papamichail et al. in the supercritical extraction of celery seed oil [26], which increased with pressure. Summarizing, the positive effects overcame the negative aspects of the extraction kinetics, and hence the yield was enhanced with pressure. The values of absolute average relative deviation (*AARD*) for both proposals indicated successful modeling of the experimental kinetic curves, and the highest value was 2.45%.

The optimized parameters from temperature variations are listed in Table S2. Like the case of pressure for both models, transition times *t*_k_ and *®t* had similar values and trends. The external mass transfer coefficients were, on average, one order of magnitude higher than the internal ones. The extraction rate was promoted when the temperature was increased. It could be demonstrated throughout the optimized *k*_f_*a* parameters, whose values also increased from the lowest to the highest temperature. The transition times tended to be shorter as the temperature was increased, resulting in higher temperatures favoring both yield and extraction rate, as was found by Wagner et al. [42]. The *AARD* resulted in a maximum of 3.55%.

Based on the particle size effect when keeping the other variables constant, it was noticed that the values for the mass transfer coefficients were in the same order of magnitude for both models, as summarized in Table S3. Values of grinding efficiency (*G* and 1 – *®x*/*x*_0_) tended to be low when particle size was increased (except for the largest particle size, which might be due to better grinding). In consequence, the experiments performed with the lowest particle size exhibited the highest yield, and hence the solubility dominated the process. On the opposite, kinetics was not so promoted by reducing the particle size because the extraction rate was slow with small particles. This trend was not the one followed by other works that studied the influence of particle size in the kinetics of supercritical fluid extraction [24]. Despite this, a higher particle size did not allow for the extraction of the majority of soluble components, which remained in the cells and were not released by grinding. The best fit correlation was obtained with the model of Sovová in comparison with the model proposed by Papamichail et al. based on the *AARD*.

Finally, the optimization of the influence of the volumetric flow rate indicated that negligible variations were found in the internal mass transfer coefficients, as well as similar values that were obtained on transition time for both models, except for the experiment performed at 3 L·min^−1^. The results for this variable are in Table S4. The coefficients *k*_f_*a* tended to decrease as the volumetric flow rate rose; this behavior was also observed in the supercritical carbon dioxide extraction of betel nut [43]. However, *e* decreased at the highest *Q_V_*. It could be probably attributed to the high velocity that avoided enough interaction between the supercritical fluid and the solutes, as the mass transfer resistance increased (low coefficients). The low values in *e* < 5% meant that these proposals were suitable for modeling the supercritical fluid extraction of *Galphimia glauca* with carbon dioxide at the studied conditions.

### 2.3. Quantification of Galphimine B

Derived from HPLC analyses, results from quantification of galphimines B and E are tabulated in Table 1, Table 2, Table 3 and Table 4. Content of galphimines were reported in terms of mg G-B·g extract^−1^, followed by expanded uncertainty with a coverage factor of 2 and relative areas for galphimine B and galphimine E. Content of galphimines in the extract was the content of the global extract, i.e., the mixture of all the extract fractions collected at the time intervals of each experiment.

The concentration of galphimine B in the extracts was within the interval from 10.4 to 29.1 mg·g^−1^, and the average concentration for all experiments was 19.5 mg·g^−1^ with a standard deviation of 5.2 mg·g^−1^. The effects of pressure, temperature, particle diameter, and solvent flow in the content of galphimines B and E were not as notorious as the variations of yields. This showed that there was uniformity in the concentration of G-B in the extracts, as well as the relative area values of galphimine E, 15.3% with 3.1% of standard deviation.

The above results on supercritical carbon dioxide extraction of G-B were comparable with those obtained by maceration of leaves and flowers of *Galphimia glauca* using different solvents in previous studies [11,12,13]. Those experiments were undertaken in ethanol:water (6:4) at 323 K for 2 h and then water:ethyl acetate (1:1) [11], water at 333 K for 2 h [12], and hexane:ethyl acetate (7:3) at 298 K for 24 h and water:ethyl acetate (1:1) [13]. Reported yields were ranged from 5.1% to 25%, while concentrations for galphimine B were reported as 1.22 [12], 50.3 [13], and 53 [11] mg·g extract^−1^. Then, the amount of the active compound, galphimine B, was found within the limits of concentration of those studies. It is important to remark that the HPLC analysis of the materials extracted with supercritical carbon dioxide showed the presence of galphimines B and E, as well as unknown compounds, whose peaks were not detected in the purified extract used as standard. This could lead to future research about the compounds in supercritical extracts and further tests, as realized by Centro de Investigación Biomédica del Sur from Instituto Mexicano del Seguro Social.

## 3. Materials and Methods

### 3.1. Material Plant

Aerial parts (leaves and flowers) of *Galphimia glauca* Cav. (Malpighiaceae) were obtained from a controlled crop developed in the State of Morelos, Mexico. MSc Abigail Aguilar Contreras performed the identification of the plant, and a voucher sample (registration number IMSSM-11061) could be found at the Medicinal Herbarium of Mexico (IMSS, Mexico City, Mexico). The material and the procedure for preparing the sample were the same as used in previous works [11,12,13]. Drying of the plant material was carried out by natural convection at room temperature and dark conditions for 14 days. Then, the dried material (leaves and flowers) was ground in an electric blender and was sieved in mesh strainers, obtaining several particle diameter fractions. Each interval (200–250, 250–425, 425–500, and 500–600 μm) consisted of 0.75 kg sample from plant material, and its particle diameter average was calculated according to ASAE S319.3 method [44], obtaining the next values: 224, 326, 461, and 548 μm. The apparent and true density of each sample listed in Table 5 was determined by a liquid displacement pycnometer [45]. These samples of the raw material of *Galphimia glauca* were stored at room temperature in sealed bags until SFE experiments.

### 3.2. Chemicals

Liquid carbon dioxide with dip tube (Coleman grade, purity 0.9999) was supplied by Infra, Mexico City, Mexico. Methanol, water, and acetonitrile (HPLC grade, purity 0.999) were purchased from Sigma-Aldrich, St. Louis, MO, USA. Ethanol and acetone (technical grade) were obtained from Fermont, Monterrey, Mexico.

### 3.3. Apparatus and Procedure

**Figure 6:**
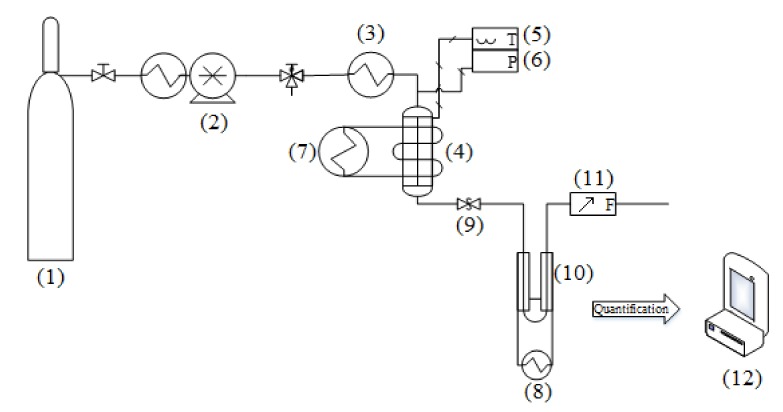
Schematic diagram of the home-made apparatus. (1) CO_2_ supply tank; (2) solvent pump; (3) preheater; (4) extractor; (5) thermometer; (6) manometer; (7,8) refrigerated circulating baths; (9) back pressure regulator; (10) U-shaped tubes; (11) wet gas meter; (12) electronic balance.

The experimental apparatus where extractions were carried out was based on the continuous method. The schematic diagram of the home-made apparatus is depicted in Figure 6.

It was mainly constituted of the carbon dioxide supply tank (1) with a dip tube, a dual-piston pump (2, model SFT-10, SFT Inc., Newark, DE, USA) with an internal Peltier chiller, an electrical coil heating tape as preheater (3), a stainless steel extraction vessel (4, model TOC7-10-GP, HiP Co., Erie, PA, USA), which is thermally controlled, a thermometer (5, model F200, ASL, Redhill, UK) coupled to a calibrated platinum resistance thermometer (100–Ω, Thermo-Est, Maizières-les-Metz, France) with an expanded uncertainty of 0.04 K, a digital manometer (6, model XP2i, Crystal pressure, San Luis Obispo, CA, USA) calibrated with an expanded uncertainty of 0.008 MPa, two refrigerated circulating baths (7,8, model PD07R, Polyscience, Niles, IL, USA), a back pressure regulator (9, model 26-1700, Tescom, St. Louis, MO, USA) to control pressure in the extractor, two U-shaped tubes arranged in series (10) immersed in a heat exchanger, and a wet gas meter (11, model WNK0.5A, Shinagawa, Tokyo, Japan). The tubular extraction vessel had an internal diameter of 2.54 cm and a length of 25.4 cm and was coiled by silicone rubber tubing connected to the liquid bath. It was internally equipped with two stainless steel filters of 5 and 2 μm screwed in the top and bottom flanges, respectively. The upper flange had a well that held the thermometer. All supercritical extraction experiments were carried out in dynamic mode and downward flow. The preparation of the SFE for each experiment started with the loading of 20 g of *Galphimia glauca* as raw material inside the extractor; then, it was filled with a layer of glass spheres (3 mm of diameter). These packed spheres and the upper filter promoted a homogeneous solvent dispersion and the mass transfer. In the beginning, the desired temperature was fixed in the preheater and the extractor, and the temperature for the last device was controlled by the refrigerated circulating bath. Besides, the temperature of the U-shaped tubes was set to 273.15 K. The thermal control in all devices was monitored up to stable conditions.

Afterward, liquid carbon dioxide was pumped from the supply tank to the top of the extractor vessel. Carbon dioxide temperature was higher than its critical temperature in the preheater located before the extractor vessel. Meanwhile, the supercritical solvent filled the extractor vessel, and the pressure was attained by the back pressure regulator, which acted as a restrictor. The extraction process was considered to begin when the desired supercritical conditions were reached.

Once the back pressure regulator was controlling pressure, the supercritical fluid flowed downwards to leave the extractor. The outer filter prevented dragging out solid particles from the fluid phase. Then, this fluid was suddenly expanded at the outlet of the back pressure regulator and allowed phase separation. In consequence, solids were precipitated in the U-shaped glass submerged in the cold container. Carbon dioxide continued flowing to the wet gas meter in order to quantify the gas volume. It was measured by considering temperature and pressure from room conditions. The mass of the extract was determined gravimetrically in an electronic balance (12, EP 520A, Precisa Gravimetrics AG, Dietikon, Switzerland).

Conversion from volumetric (flow rate and total volume) to mass units for carbon dioxide was performed using the Equation of State published by Span and Wagner [46]. The mass of the extract at each time was registered and added to the previous measurement, in order to obtain cumulative extract mass. Each tube was washed with methanol and placed in a single flask to analyze the total extract on the SFE condition. Samples were stored in sealed vials and kept cooled until further analysis. Every experiment was stopped when there was no significant variation of mass between each register. Most of the experiments took an extraction time of 240 min, with a range from 90 to 390 min.

### 3.4. Analyses

Quantification of galphimine B was performed by means of HPLC chromatography (model series 200, Perkin Elmer, Waltham, MA, USA), based on previous studies [9]. The methanolic solutions previously filtered were injected throughout a 20 μL sampling loop. A gradient of acetonitrile:water (A:W) as a mobile phase was used for the separation of components at a constant flow rate of 0.9 mL/min in a Spheri-5 RP-18 5 μm column 250 × 4.6 mm equipped with a guard column with the same stationary phase. A volume ratio of 40:60 was fixed for the first 10 min, the ratio was changed to 52:48 after 1 min and kept for 4 min; then, it was changed to 70:30 at 16 min, 88:12 at 17 min, and 94:6 at 18 min maintained for 2 min; 100% acetonitrile was reached at 22 min and kept for 1 min. Finally, a ratio of 40:60 was fixed at 25 min, and the analysis was stopped at 35 min. The absorbance of galphimine B was set to 230 nm in the UV-Vis detector.

A purified extract of galphimines with the known composition of galphimine B (G-B) was used as a standard for the detector calibration, which was obtained with the procedure described by Romero-Cerecero et al. [11]. This standard is constituted mainly by galphimines B and E, and the concentration of galphimine B is 0.2 mg·g^−1^. From this standard, the unknown composition of galphimine E was also reported as a relative area. A calibration curve with galphimine B was attained using concentrations of 1, 5, 10, 15, 20, 25, 30, 35 μg G-B/mL methanol. Results were adjusted to a linear function: *C*_G-B_ = 42.3341*AU*_G-B_ – 0.6819, where *C*_G-B_ is the concentration of G-B in μg/mL, and *AU*_G-B_ is the response area of G-B. In the case of galphimine E, the quantification was limited to as the percentage of the relative area between galphimine E and galphimine B.

### 3.5. Mathematical Modeling

Modeling of supercritical fluid extraction curves was achieved based on models developed with mass balances in the fluid and solid phases, reported elsewhere [24,26]. One of the most applied models is the one proposed by Sovová, which was developed originally for representing the extraction of oils from milled seeds by rejecting the interactions between the solid and fluid phases. This model is expressed in Equation (1) [47] as follows:

(1) $$\frac{E}{Nx_{0}}=\left\{\begin{array}{ccc} \Psi \left(1-e^{-Z}\right) & \mathrm{for} & \Psi <\frac{G}{Z}\\ \Psi -\frac{G}{Z}e^{Z\left(h_{k}-1\right)} & \mathrm{for} & \frac{G}{Z}\leq \Psi <\Psi_{k},\\ 1-\frac{1}{Y}\ln\left[1+\left(e^{Y}-1\right)e^{\left[Y\left(\frac{G}{Z}-\Psi \right)\right]}\left(1-G\right)\right] & \mathrm{for} & \Psi \geq \Psi_{k} \end{array}\right.,$$

where the dimensionless variables *G*, *Z*, *Y*, *Ψ*, *Ψ*_k_, and *h*_k_ are defined by the next expressions: *G* = 1 – *x*_k_/*x*_0_, *Z* = *Nk*_f_*aρ*_f_/*Q*(1 – *ε*)*ρ*_s_, *Y* = *Nk*_s_*ax*_0_/*Q*(1 – *ε*)*y*_r_, *Ψ* = *tQy*_r_/*Nx*_0_, *Ψ*_k_ = *G*/*Z* + (1/*Y*) ln{1 – *G* [1 – exp(*Y*)]}, and *h*_k_ = (1/*Y*) ln[1 + {exp[*Y*(*Ψ* – *G*/*Z*)] – 1}/*G*]. The above expressions include additional variables, which comprise physical meaning. *E* is referred to the mass of extracted solute, *N* denotes the mass of the solid material, *x*_0_ corresponds to the initial concentration of solute in the solid phase, *x*_k_ represents the interface concentration of solute, *t* symbolizes the extraction time, *Q* indicates the mass flow rate of the supercritical fluid, *ε* expresses the bed porosity, *a* is the interfacial area, *ρ*_f_ represents the supercritical fluid density, *ρ*_s_ is referred to the solid density, *k*_f_ describes the external mass transfer coefficient, which is optimized together with the interfacial area *a* as the product *k*_f_*a*, *k*_s_ denotes the internal mass transfer coefficient, and *y*_r_ corresponds to the solute solubility in the supercritical fluid as apparent parameter in multicomponent matrixes, which actually is the apparent solubility, as it was not experimentally determined. Apparent solubility was obtained on each experimental condition as the slope of a plot of the cumulative mass of extract versus the mass of CO_2_ spent per mass of material charged in the extractor.

The model of Sovová is adequate for modeling the supercritical fluid extraction of vegetable oils that commonly produces high yields (*e* > 10%). To the best of our knowledge on applications with low yields, the mass transfer equilibrium is usually controlled by the specific interactions between the solute (solid phase) and the supercritical solvent (fluid phase). This is the case for the results obtained in this work since the highest yield was reported to be less than 2.22%. Therefore, the approach proposed by Papamichail et al. [20] was also applied. A remarkable characteristic of this model is the consideration of those specific interactions between the solute and solvent. The expressions for this model are summarized as follows:

(2) $$\begin{array}{c} e=\left\{\begin{array}{ccc} y_{0}A\left(1-B\right)t & \mathrm{for} & x\geq \bar{x}\;\mathrm{or}\;t\leq \bar{t}\\ x_{0}-\bar{x}e^{-AK\left(1-B\right)\left(t-\bar{t}\right)} & \mathrm{for} & x<\bar{x}\;\mathrm{or}\;t>\bar{t} \end{array}\right. \end{array},$$

where *e* corresponds to the extraction yield, *y*_0_ denotes the solute solubility in the supercritical fluid similar to *y*_r_ from Equation (1), *A* is a variable that comprises the overall mass transfer coefficient in the fluid phase (*k*_f_*a*) and is defined as *k*_f_*aρ*_f_/*ρ*_s_(1–*ε*), and *B* is quantified by *A*/(*Q̇* + *A*). *Q̇* is expressed as the specific mass flow rate of the supercritical fluid estimated with the *Q*/*N* ratio. These additional definitions can state that *A* is equivalent to the product *ZQ̇*, as well as *®x* is also equivalent to *x*_k_ reported in the equations proposed by Papamichail et al. and Sovová, in that order.

The model proposed by Papamichail et al. in Equation (2) states that the extraction is controlled by two stages, each one dominated by solubility and diffusion mechanisms. The first expression for yield is applied when *x* ≥ *®x* and indicates that the extraction is under the solubility controlled regime, while the second expression is utilized when *x* < *®x* and signifies that the process is subjected to a diffusion-controlled regime. The boundary for each regime is given by either the solute concentration in the solid phase *®x* or the time *®t*. This latter variable is defined as (*x*_0_ – *®x*)/[(*y*_0_*A*(1 – *B*)]. Moreover, *K* is the equilibrium constant in Equation (3) that was considered from Perrut et al. [48]:

(3) $$\begin{array}{cc} y^{*}=y_{0} & x\geq \bar{x}\\ y^{*}=Kx & x<\bar{x} \end{array},$$

Sovová defines *Ψ*_k_ as the dimensionless time that is the boundary between the easy and difficult extraction stages. Then, the time of the boundary *t*_k_ can be obtained from *Ψ*_k_. A comparable assumption with *t*_k_ for the Papamichail et al. model is that *®t* has the equivalent meaning. In the same way, the grinding efficiency *G* is equivalent to 1 – *®x*/*x*_0_.

Parameters for both models were obtained by minimizing the objective function based on the yield in Equation (4):

(4) $$F_{\mathrm{obj}}=\sum_{i=1}^{i=n}{\left(e_{i}^{\exp}-e_{i}^{\mathrm{calc}}\right)}^{2},$$

The absolute average relative deviation (*AARD*) was calculated with Equation (5):

(5) $$AARD=\frac{100}{n}\sum_{i=1}^{i=n}\left|1-\frac{e_{i}^{\mathrm{calc}}}{e_{i}^{\exp}}\right|,$$

In Equations (4) and (5), *n* denotes the number of data points, and *e_i_* refers to the yield for each experiment. Superscripts exp and calc correspond to the experimental and calculated data points, respectively.

## 4. Conclusions

Supercritical carbon dioxide extraction from *Galphimia glauca* was carried out in a home-made apparatus. The influence of *P*, *T*, *d*_p_, and *Q_V_* on the extracts was studied in a wide range, achieving maximum yield of 2.22% in dry basis. High pressure favored higher yields as the solvent density was superior and allowed the increase of its solvation power and the capacity for dissolving more substances. The values of extraction yields suggested variations in parameters, such as static period previous to dynamic mode, as well as the evaluation of a polar co-solvent, due to the presence of multiple phenolic and high molecular weight compounds. The kinetic curves were represented with good results by the Sovová and Papamichail et al. equations. Successful extraction of galphimine B was attained based on the content of this nor-seco triterpenoid in the extract, whose concentration was comparable with those results via the maceration technique reported previously. The variations of galphimine B content as a function of *P*, *T*, *d*_p_, and *Q_V_* was almost negligible; it could probably be associated with the preference of carbon dioxide to dissolve other chemicals among galphimine B and galphimine E.

## Figures and Tables

**Table 1:** Galphimines and yield from supercritical carbon dioxide extraction of *Galphimia glauca* at temperature = 323.15 K, particle size = 326 μm, and volumetric flow rate = 3 L·min^−1^.

*P* (MPa)	*e* (%)	Concentration G-B (mg·g extract^−1^)	Relative area G-B (%)	Relative area G-E (%)
15.00	0.70	18.1 ± 2.3	85.24	14.76
21.25	1.55	28.1 ± 8.3	88.06	11.94
27.50	1.82	21.6 ± 1.5	86.11	13.89
33.75	2.22	20.3 ± 2.6	86.02	13.98

**Table 2:** Galphimines and yield from supercritical carbon dioxide extraction of *Galphimia glauca* at pressure = 27.50 MPa, particle size = 326 μm, and volumetric flow rate = 3 L·min^–1^.

*T* (K)	*e* (%)	Concentration G-B (mg·g Extract^−1^)	Relative Area G-B (%)	Relative Area G-E (%)
313.15	0.92	11.5 ± 2.6	91.38	8.62
318.15	0.79	29.1 ± 2.2	84.92	15.08
323.15	1.82	21.6 ± 1.5	86.11	13.89
328.15	1.96	11.7 ± 1.2	80.49	19.51

**Table 3:** Galphimines and yield from supercritical carbon dioxide extraction of *Galphimia glauca* at pressure = 27.50 MPa, temperature = 323.15 K, and volumetric flow rate = 3 L·min^–1^.

*d*_p_ (μm)	*e* (%)	Concentration G-B (mg·g Extract^−1^)	Relative Area G-B (%)	Relative Area G-E (%)
224	2.16	21.4 ± 2.7	79.56	20.44
326	1.82	21.6 ± 1.5	86.11	13.89
461	1.76	17.5 ± 1.8	79.54	20.46
548	0.78	19.0 ± 2.3	84.13	15.87

**Table 4:** Galphimines and yield from supercritical carbon dioxide extraction of *Galphimia glauca* at pressure = 27.50 MPa, temperature = 323.15 K, and particle size = 326 μm.

*Q_V_* (L·min^−1^)	*e* (%)	Concentration G-B (mg·g Extract^−1^)	Relative Area G-B (%)	Relative Area G-E (%)
1	0.91	19.5 ± 1.5	83.56	16.44
2	1.30	19.1 ± 3.1	85.79	14.21
3	1.82	21.6 ± 1.5	86.11	13.89
4	1.40	10.4 ± 1.3	82.31	17.69

**Table 5:** Particle diameter and density of *Galphimia glauca* samples.

Average *d*_p_ (μm)	Apparent Density (kg·m^−3^)	True Density (kg·m^−3^)
224	291	1157
326	266	1117
461	250	1091
548	194	994

## Supplementary Materials

The following are available online. Table S1: Modeling results for supercritical fluid extraction of *Galphimia glauca* at *T* = 323.15 K, *d*_p_ = 326 μm, and *Q_V_* = 3 L·min^−1^. Table S2: Modeling results for supercritical fluid extraction of *Galphimia glauca* at *P* = 27.50 MPa, *d*_p_ = 326 μm, and *Q_V_* = 3 L·min^−1^. Table S3: Modeling results for supercritical fluid extraction of *Galphimia glauca* at *P* = 27.50 MPa, *T* = 323.15 K, and *Q_V_* = 3 L·min^−1^. Table S4: Modeling results for supercritical fluid extraction of *Galphimia glauca* at *P* = 27.50 MPa, *T* = 323.15 K, and *d*_p_ = 326 μm.

## Abbreviations

*A*	Variable in the model of Papamichail et al. (min^−1^)
*AU*_G-B_	Response area of galphimine B (absorbance^2^)
*a*	Interfacial area (m^−1^)
*B*	Variable in the model of Papamichail et al. (dimensionless)
*C*_G-B_	Concentration of galphimine B (μg·mL^−1^)
*d*_p_	Particle size (μm)
*E*	Mass of extract (g)
*e*	Extraction yield (dimensionless)
*G*	Grinding efficiency (dimensionless)
*h*	Coordinate in the model of Sovová (dimensionless)
*K*	Equilibrium constant in the model of Papamichail et al. (dimensionless)
*k*	Mass transfer coefficient (m·min^−1^)
*N*	Mass of solid charged in the extractor (g)
*n*	Number of datum
*P*	Pressure (MPa)
*Q*	Mass flow rate (g·min^−1^)
*Q_V_*	Volumetric flow rate (L·min^−1^)
*T*	Temperature (K)
*t*	Time (min)
*x*	Concentration in the solid phase (dimensionless)
*Y*	Variable in the model of Sovová (dimensionless)
*y*	Concentration in the fluid phase (dimensionless)
*y*_0_	Solubility in the model of Papamichail et al. (g_extract_·g_CO2_^−1^)
*y*_r_	Solubility in the model of Sovová (g_extract_·g_CO2_^−1^)
*Z*	Variable in the model of Sovová (dimensionless)
Greek letters	
*ε*	Bed porosity (dimensionless)
*Ψ*	Dimensionless time in the model of Sovová (dimensionless)
ρ	Density (kg·m^−3^)
Superscripts	
®	Boundary of extraction periods in the model of Papamichail *et al*.
*	Equilibrium condition
˙	Specific variable
calc	Calculated
exp	Experimental
Subscripts	
0	Initial condition
f	Fluid phase
k	Boundary of extraction periods in the model of Sovová.
s	Solid-phase
