# Supplementary material for: Extraction of Galphimines from Galphimia glauca with Supercritical Carbon Dioxide
Source: Molecules. 2020 Jan 22;25(3):477. doi: 10.3390/molecules25030477 (PMC7037395; doi:10.3390/molecules25030477)
Supplement: Supplementary file 1 [file molecules-25-00477-s001.pdf]

Supplementary materials

Extraction of Galphimines from *Galphimia glauca* with Supercritical Carbon Dioxide

Francisco Javier Verónico Sánchez [1], Octavio Elizalde Solis [1,*], Alejandro Zamilpa [2], Ricardo García Morales [1], Ma. Dolores Pérez García [2], Jesús E. Jiménez Ferrer [2] and Jaime Tortoriello [2]

1 Departamento de Ingeniería Química Petrolera and Sección de Estudios de Posgrado e Investigación, Escuela Superior de Ingeniería Química e Industrias Extractivas, Instituto Politécnico Nacional (IPN), 07738 Mexico City, Mexico
2 Centro de Investigación Biomédica del Sur, Instituto Mexicano del Seguro Social (CIBIS-IMSS), 62790 Morelos, Mexico
* Correspondence: oelizalde@ipn.mx or octavioel@yahoo.com.mx; Tel.: +52-55-5729-6000 (ext. 55120 or 55124)

Table S1. Modelling results for supercritical fluid extraction of *Galphimia glauca* at T = 323.15 K, d_p = 326 μm and Q_V = 3 L·min^{-1}.

		Sovová [24]					Papamichail et al. [26]				
P **(MPa)**	$y_r \cdot 10^3$ ($g_{extract} \cdot g_{CO2}^{-1}$)	$k_{fa} \cdot 10^2$ (min^{-1})	$k_{sa} \cdot 10^3$ (min^{-1})	G (–)	t_k (min)	*AARD* (%)	$k_{fa} \cdot 10^2$ (min^{-1})	K (–)	$1-\bar{x}/x_0$ (–)	$\bar{t}$ (min)	*AARD* (%)
15.00	0.753	23.001	6.807	0.52	39.33	1.55	27.208	0.12	0.53	27.19	1.44
21.25	1.889	58.751	4.053	0.52	28.40	2.11	72.928	0.07	0.53	23.86	2.45
27.50	2.282	2.498	8.190	0.52	83.71	0.67	0.851	0.34	0.53	69.32	1.21
33.75	3.754	1.324	8.574	0.52	95.06	1.93	0.415	0.61	0.53	85.32	2.12

Table S2. Modelling results for supercritical fluid extraction of *Galphimia glauca* at P = 27.50 MPa, d_p = 326 μm and Q_V = 3 L·min^{-1}.

		Sovová [24]					Papamichail et al. [26]				
T **(K)**	$y_r \cdot 10^3$ ($g_{extract} \cdot g_{CO2}^{-1}$)	$k_{fa} \cdot 10^2$ (min^{-1})	$k_{sa} \cdot 10^3$ (min^{-1})	G (–)	t_k (min)	*AARD* (%)	$k_{fa} \cdot 10^2$ (min^{-1})	K (–)	$1-\bar{x}/x_0$ (–)	$\bar{t}$ (min)	*AARD* (%)
313.15	0.556	11.691	9.898	0.52	95.40	2.27	7.958	0.15	0.53	57.81	1.52
318.15	1.242	2.852	6.551	0.52	57.65	3.55	0.987	0.28	0.53	48.02	3.26
323.15	2.282	2.498	8.190	0.52	83.71	0.67	0.851	0.34	0.53	69.32	1.21
328.15	5.699	23.801	5.592	0.52	12.75	1.97	25.242	0.09	0.53	10.41	2.67

Table S3. Modelling results for supercritical fluid extraction of *Galphimia glauca* at P = 27.50 MPa, T = 323.15 K and Q_V = 3 L·min^{-1}.

		Sovová [24]					Papamichail et al. [26]				
d_p (μm)	$y_r \cdot 10^3$ ($g_{extract} \cdot g_{CO2}^{-1}$)	$k_{fa} \cdot 10^2$ (min^{-1})	$k_{sa} \cdot 10^3$ (min^{-1})	G (–)	t_k (min)	*AARD* (%)	$k_{fa} \cdot 10^2$ (min^{-1})	K (–)	$1-\bar{x}/x_0$ (–)	$\bar{t}$ (min)	*AARD* (%)
224	2.282	4.944	4.305	0.89	105.02	2.13	1.831	0.10	0.88	85.51	2.11
326	2.282	2.498	8.190	0.52	83.71	0.67	0.851	0.34	0.53	69.32	1.21
461	2.282	1.352	7.869	0.41	56.71	2.90	0.307	0.47	0.25	54.09	3.67
548	2.282	1.599	6.075	0.86	59.02	1.85	0.334	0.48	0.87	51.83	1.83

Table S4. Modelling results for supercritical fluid extraction of *Galphimia glauca* at P = 27.50 MPa, T = 323.15 K and d_p = 326 μm.

Q_V (MPa)	$y_r \cdot 10^3$ ($g_{extract} \cdot g_{CO2}^{-1}$)	Sovová [24]					Papamichail et al. [26]				
		$k_{fa} \cdot 10^2$	$k_{sa} \cdot 10^3$	G	t_k	*AARD*	$k_{fa} \cdot 10^2$	K	1 –	$\bar{t}$	*AARD*

		**(min⁻¹)**	**(min⁻¹)**	**(–)**	**(min)**	**(%)**	**(min⁻¹)**	**(–)**	$\bar{x}/x_0$ **(–)**	**(min)**	**(%)**
1	2.282	54.753	6.083	0.52	39.30	2.77	58.153	0.31	0.53	30.97	4.12
2	2.282	14.599	6.908	0.52	35.44	3.02	15.004	0.18	0.53	25.62	3.84
3	2.282	2.498	8.190	0.52	83.71	0.67	0.851	0.34	0.53	69.32	1.21
4	2.282	6.463	6.223	0.52	30.73	3.25	2.652	0.14	0.53	24.41	3.55
